# Coronary Sinus Stenting for the Management of Left Ventricular Lead Displacement during Resynchronization Therapy: A Report of Two Cases

**Published:** 2018-01

**Authors:** Hassan Kamalzadeh, Shahrooz Yazdani, Mohammad Jalali

**Affiliations:** *Shahid Rajaei Hospital, Alborz University of Medical Sciences, Karaj, Iran.*

**Keywords:** *Cardiac resynchronization therapy*, *Stents*, *Angioplasty*, *Coronary sinus*

## Abstract

In patients with cardiac resynchronization therapy (CRT), loss of left ventricular (LV) stimulation occurs chiefly because of LV lead dislodgement. The occurrence rate of LV lead dislodgement in different reports is between 2% and 12% of patients. LV lead dislodgement precludes clinical improvement. We describe 2 patients with heart failure, fulfilling the criteria for CRT implantation. In both patients, right ventricular and right atrial leads were implanted via the left subclavian vein in the right ventricular apex and the right atrial appendage, respectively. Repeated LV lead implantation was unsuccessful and each time after the fixation, the LV lead was dislodged with the heart motion during systole and diastole. In order to stabilize the LV lead, we decided to benefit from coronary sinus stenting and lead entrapment behind the deployed stent. LV lead stabilization was accomplished by the deployment of bare-metal stents (Multi-Link 3.5 × 8 mm and Multi-Link 3 × 8 mm, Abbott Vascular) in order to entrap the LV lead. The stents were deployed at a nominal pressure (10 atm). The pacing performance of the LV leads was satisfactory and stable at midterm in our experience. Stenting within the coronary sinus seems to be a safe method for LV lead stabilization and can substantially boost the success rate of CRT. Our device analysis during short- and midterm follow-up (4 months after implantation) revealed acceptable LV lead threshold and impedance.

## Introduction

Cardiac resynchronization therapy (CRT), also known as biventricular pacing or multisite ventricular pacing, involves simultaneous or different-time pacing of the right ventricle (RV) and the left ventricle (LV). To this end, a coronary sinus (CS) lead is placed for LV pacing in addition to a conventional RV endocardial lead. Clinical trials have established the benefits of CRT in the treatment of patients suffering from heart failure. As adjuncts to guideline-directed medical therapy, CRT reduces mortality and hospitalization rates and improves functional capacity. The basic goal of CRT is to restore LV synchrony in patients with cardiomyopathy and a widened QRS, which is predominantly a result of left bundle branch block (LBBB), with a view to augmenting the mechanical functioning of the LV.^1^

CRT is a valuable procedure in appropriate subgroups of patients with heart failure. CRT is mostly beneficial for patients suffering from heart failure with LV ejection fractions less than 35% and intraventricular conduction delay (mainly left bundle branch block [LBBB]).^2^^-^^4^ When combined with an implantable defibrillator, CRT can enhance both functional capacity and survival.^5^

OpenUrOpenUrlODuring CRT implantation, LV lead placement in the appropriate CS vein branch with the suitable size may be challenging.^6^ Additionally, in circumstances where the LV lead is not fixed in a stable position, early lead displacement with resultant resynchronization failure may ensue.^7^ Early LV lead displacement is reported in about 5% of procedures.^8^ Nevertheless, collaboration between physicians and industry in tandem with enhanced experience of electrophysiologists has decreased the implantation failure rate from 8% to 2%.

OpenUrlLV lead displacement leads to loss of biventricular pacing and ineffectiveness of CRT implantation. We herein describe 2 patients with difficulty in LV lead fixation, which was resolved with an unusual and off–label technique.

## Case Reports

***Case # 1***

A 61-year-old man with a history of myocardial infarction and previous coronary artery bypass grafting many years previously was admitted due to severe heart failure symptoms unresponsive to optimal medical therapy. At admission, the patient was in sinus rhythm with LBBB (QRS duration = 160 ms) in the New York Heart Association (NYHA) functional class III. The LV ejection fraction in echocardiography was 20%.

**Figure 1:**
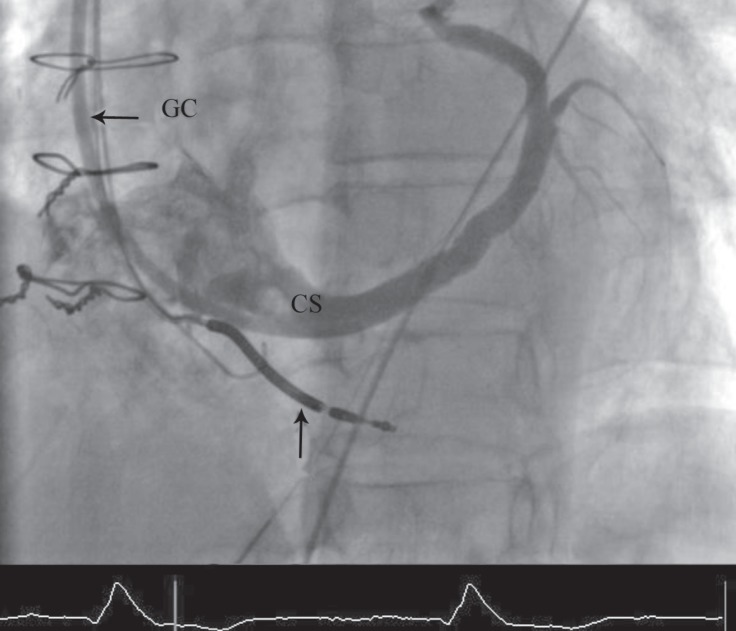
Venography of the coronary sinus (CS) in the shallow left anterior oblique projection. During the process of cardiac resynchronization therapy, injection in the CS via the guiding catheter (GC) illustrates the CS anatomy for appropriate CS lead placement. The right ventricular lead is shown with the vertical arrow.

An RV lead (Durata 7122-65, St. Jude Medical) and a right atrial lead (Tendril STS-2088TC-52) were implanted in the RV apex and the right atrial appendage, respectively, via the left subclavian vein. Subsequently, a CS delivery system was introduced into the CS through the left subclavian vein. CS angiography revealed an unsuitable venous branch in the lateral area (Figure 1), but the posterolateral branch had an acceptable size and anatomy. Accordingly, an LV lead (QuickFlex 1258T-86 with a 5 F body diameter) was implanted at the distal portion of the posterolateral vein branch. Nevertheless, the LV lead had no stable fixation and subsequent lead dislodgement occurred shortly after implantation.

**Figure 2:**
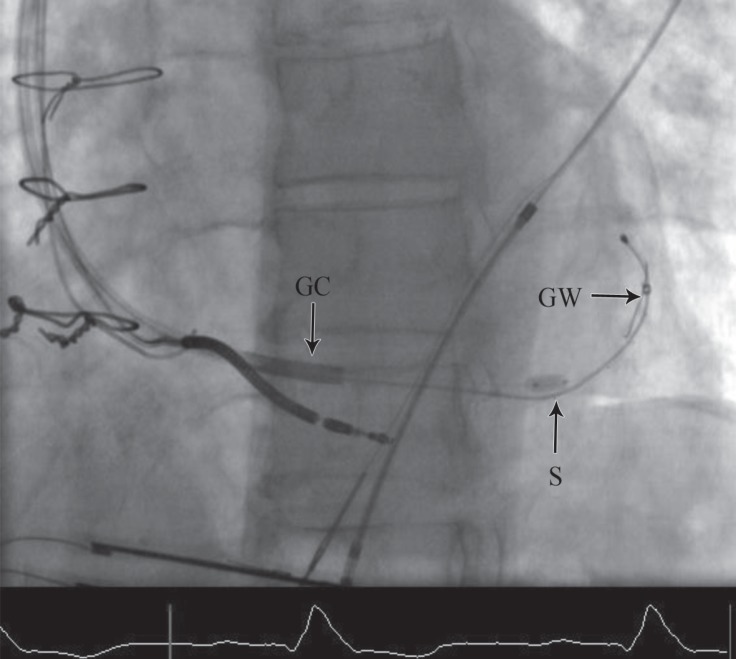
Coronary sinus (CS) lead stabilization with stenting is shown in the shallow left anterior oblique projection. The coronary sinus lead and a 0.014-inch guide wire are both passed through the guiding catheter. The CS lead is stabilized and entrapped by stenting the CS.

GC, Guiding catheter; GW, Guide wire; S, Stent development

Repeated LV lead implantation was unsuccessful and each time after the fixation, the LV lead was displaced with the heart motion during systole and diastole. The LV lead was stabilized in the posterolateral vein branch of the CS via CS stenting and lead entrapment behind the deployed stent. A 0.014-inch coronary guide wire (HI-TORQUE PILOT 50) was advanced into the posterolateral vein branch of the CS through the delivery system. A bare-metal stent (Multi-Link 3 × 8 mm, Abbott Vascular) was advanced over the wire and placed parallel to the LV lead. The stent was deployed at a nominal pressure (10 atm) 3 cm distal to the venous branch takeoff, and the distal part of the stent was 1 cm before the LV ring (Figure 2). After CS stenting, the delivery system was removed safely without LV lead dislodgement. Our device analysis during short- and midterm follow-up (4 months after implantation) revealed acceptable LV lead threshold and impedance (0.5 V and 1070 Ω, correspondingly).

***Case # 2***

**Figure 3:**
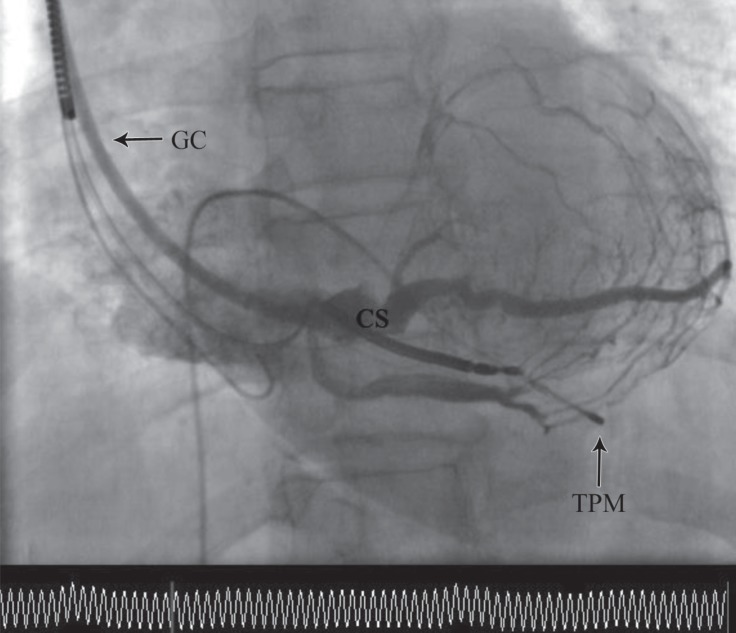
Coronary sinus (CS) venography in the shallow left anterior oblique projection. CS injection is performed via a guiding catheter to clarify the appropriate site for CS lead placement.

GC, Guiding catheter; TPM, Temporary pacemaker

**Figure 4:**
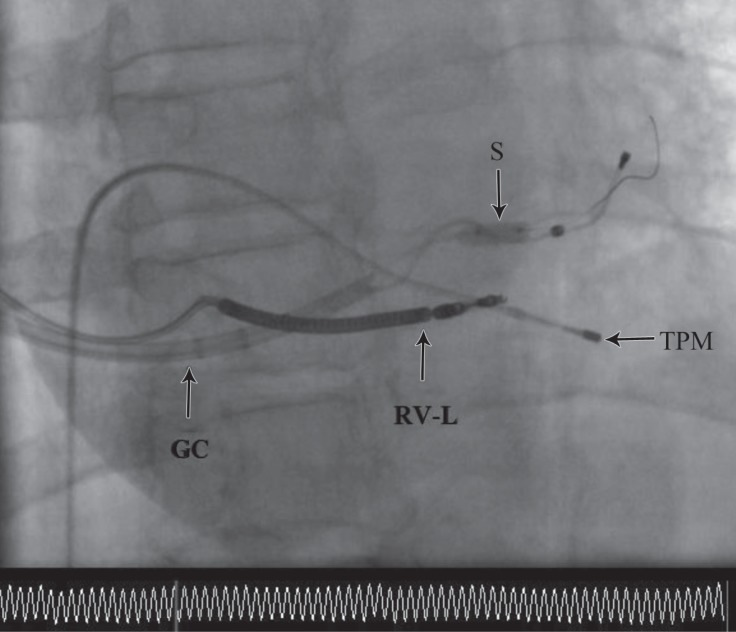
Stent deployment to stabilize the coronary sinus (CS) lead in the right anterior oblique projection. In this image, CS lead stabilization is shown with stent deployment.

GC, Guiding catheter; RV_L, Right ventricle lead; S, Stent development; TPM, Temporary pacemaker

A 71-year-old woman with a history of ischemic cardiomyopathy with an LV ejection fraction of about 20% and the NYHA functional class III who was symptomatic despite optimal medical therapy was admitted for cardiac resynchronization therapy defibrillator (CRT-D) implantation. Her electrocardiography was in sinus rhythm with an LBBB morphology (QRS duration = 160 ms). An RV lead (Durata 7122-65, St. Jude Medical) and a right atrial lead (Tendril STS-2088TC-52) were implanted into the RV apex and the right atrial appendage, respectively, via the left subclavian vein. After CS venography, an LV lead (QuickFlex 1258T-86) was implanted in the lateral marginal branch, but the procedure was unsuccessful due to the small size of the venous branch (Figure 3). Consequently, the LV lead was repositioned in the posterolateral branch of the CS. A few minutes later, spontaneous lead displacement occurred. LV lead stabilization was accomplished by the deployment of a bare-metal stent (Multi-Link 3.5 × 8 mm, Abbott Vascular) about 4 cm before the LV lead ring in order to entrap it (Figure 4). The baseline and midterm follow-up at 6 months showed acceptable LV lead pacing threshold (0.5 and 0.5 V) and impedance (550 and 780 Ω), respectively.

## Discussion

CRT is an effective treatment in patients with severe refractory heart failure combined with intraventricular conduction disease inasmuch as it improves quality of life and decreases mortality. In CRT, pacing of the LV is accomplished with a CS electrode. The main challenge in this technique is to achieve and maintain an optimal lead position so that no dislocation occurs.^9^^, ^^10^

The effectiveness of CRT is significantly hindered in 30% of recipients for several reasons. Aside from the issue of appropriate patient selection, loss of LV stimulation plays an important role. Loss of LV stimulation occurs mainly because of LV lead dislodgement, which is reported to range from 2% to 12% of patients in different reports.^11^^, ^^12^

In cases of LV lead implantation failure, the surgical approach for epicardial lead implantation is a well-known option. This approach, however, imposes further risk on the patient and increases both morbidity and mortality in comparison with the conventional transvenous approach. Ailawadi and colleagues^13^ showed that surgical LV lead placement conferred functional benefits similar to those of percutaneous placement but with greater risk of perioperative complications, including acute renal failure and infection. Koos et al.^14^ demonstrated that the surgical approach for LV lead placement in CRT systems led to higher mortality at 1 year’s follow-up than the transvenous approach. Therefore, CS leads are preferable to epicardial leads. In a study by Dekker et al.,^15^ surgical LV lead implantation increased mortality and morbidity, including acute renal failure, from 4.9% to 26%. In the surgical arm, the postprocedural infection rate also rose from 2.4% to 11.9%. In another study by Poole et al.,^16^ among 434 patients for CRT implantation, 48 cases had failed transvenous procedures. Considering this relatively high failure rate, it is advisable that feasible alternatives be emphasized in cases of LV lead placement failure. What should also be borne in mind, however, is that delivery systems for the CS cannulation and accessory devices in cases of lead placement failure are limited.

CS venoplasty with stent placement has been performed in patients in whom LV lead advancement fails owing to CS occlusion or dissection or vein tortuosity.^9^ In some cases, CRT implantation can be difficult, particularly when seeking to establish optimal LV stimulation through proper lead placement. Difficulties can arise from venous stenosis, atypical and tortuous CS anatomy, presence of venous valves, postoperative deformation, and absence of vessels in the target location. Various methods adapted from percutaneous coronary artery interventions can be applied to resolve these problems and ensure a good lead position.^17^

Luedorff and colleagues^18^ showed that angioplasty of the coronary or subclavian veins and the CS valve structures is a useful and safe tool for successful lead placement. The use of balloons, 3.0 mm in size, usually allows implantation of at least a unipolar lead. 

Angioplasty of the CS branches during CRT implantation procedures bears the risk of complete branch occlusion, but recanalization can be acutely achieved by stent implantation. Gutleben et al.^19^ reported the first case on rescue-stenting of a CS branch after angioplasty-related occlusion. 

Although CS stenting for LV lead stabilization has been reported previously, this approach is not widely accepted due to concerns about probable insulation defects after stent deployment near the silicon cover of the LV lead.^20^ In contrast to the previous reports, which used a separate subclavian vein puncture and a second catheter (guiding catheter) to deliver the stent, we advanced the coronary guide wire and stent via the same CS delivery system catheter.

Oto and colleagues^21^ attempted transvenous LV lead implantation for CRT in 255 patients, 6.7% of whom needed CS intervention. In that study, CS angioplasty was performed in 16 (6.2%) and stenting in 3 (1.2%) patients to facilitate LV lead placement. Additionally, 2 patients needed both balloon angioplasty and stenting. LV leads were successfully inserted in 15/17 (88.2%) of the patients who needed CS intervention. There were no complications related to CS intervention. The authors reported that the overall success rate of LV lead implantation was increased from 238/255 (93.3%) to 253/255 (99.2%) by the use of CS intervention.

The pacing performance of the LV leads in the series by Geller et al.^22^ was satisfactory and stable at long term. In in their experience, they were able to demonstrate only minor surface damage of the stent on the lead insulator.

Almost all LV leads can be maintained in coronary veins with satisfactory safety. If a small (5 F or lesser diameters) lead is delivered via a typical 8 F guide catheter, stents up to 4 mm can be delivered via the same guide while retaining the lead. In contrast to the stents used for dissection, retention stents should be smaller by 0.5 to 1 mm than the vein diameter at the site of deployment because the only objective of the stent is to grant resistance to lead dislodgment. If the stent is oversized or improperly long, the lead may be trapped such that it cannot be withdrawn without using more invasive techniques. It is preferable to deploy the stent as adjacent to the pacing electrode as possible for maximal constancy. In practice, this is usually the tip electrode. Nonetheless, the stent should not make contact with the cathode because that would increase the surface area of the cathode and result in an increase in the pacing threshold. It should be acknowledged that the use of stents for active LV lead fixation is a novel technology, which is not advocated on a routine basis until randomized trials demonstrate their safety. There is concern, for example, regarding the possibility of damage to the insulation or the conductor of the lead. This seems unlikely, given that coronary artery stents are flexible objects designed to accommodate the coronary artery anatomy and to move without causing harm. Coronary venous stenting can be safe and helpful if performed by properly trained physicians. Familiarity with interventional tools and techniques is essential for success. The following points should be kept in mind while employing these techniques:

Only bare-metal stents of proper lengths and calibers for the application should be deployed. The only likely exception is the deployment of a long stent in a low-flow main CS, where the occlusion of the stent by thrombi may have severe consequences. In such cases, a drug-eluting stent may be chosen, but appropriate antiplatelet therapy will then be required. Our experience in this regard is limited, and there are no published data to support or disprove this belief.

For lead retention, the shortest available stent should be deployed. It should be approximately 0.5 mm smaller than the noticeable vein diameter because the vein will accommodate the stent as well as the lead. This successfully retains the lead in position while allowing the withdrawal of the lead over a stiff guide wire if necessary.

We have previously had an experience of CS angioplasty for coronary vein stenosis and LV lead insertion for CRT implantation, which was published as a case report in 2007.^23^ In our procedure, we drew upon the CS delivery system to guide us to the main trunk of the CS. Afterward, we delivered the guide wire to the target (CS branch) and sent the balloon catheter (over the wire) to the appropriate position. Finally, we deployed the stent and entrapped the LV lead in the suitable position. We did not use separate conventional guiding catheters, which are used in percutaneous coronary interventions.

## Conclusion

In light of our experience, coronary sinus stenting for the stabilization of the left ventricular lead is technically feasible with a satisfactory midterm follow-up.

## Notes:

***This paper should be cited as:*** Kamalzadeh H, Yazdani S, Jalali M. Coronary Sinus Stenting for the Management of Left Ventricular Lead Displacement during Resynchronization Therapy: A Report of Two Cases. J Teh Univ Heart Ctr 2018;13(1):27-31.
